# QOI7/423: Does Giving Patient Information by Internet Make Sense?

**DOI:** 10.2196/jmir.1.suppl1.e100

**Published:** 1999-09-19

**Authors:** JW Van der Slikke

**Affiliations:** ^1^Department of Obstetrics & Gynaecology, ZaandamThe Netherlands

**Keywords:** Internet, Information, Education;

## Abstract

**Introduction:**

In the European collaboration "WOMAN"-project there is amongst others a set up for patient information and education about menopause on the WWW. One of the questions was how many women in Europe have access to Internet.

**Methods:**

In our clinic (Department of Obstetrics and Gynaecology of the "de Heel" hospital) we used the WOMAN-questionnaire. In this form questions are asked about computer use (at work and at home), if the respondent knew Web sites that gave information about menopause and if not, if they were interested in such Web sites. Each consecutive woman, born between 1940 and 1960 visiting our outpatient clinic received a questionnaire. We asked her to complete the form while she was waiting for the doctor. Because one of our theories was, that Internet-use is age-dependent, we created a second group: We developed a form, in the same format as the menopause-form for pregnant women.

**Results:**

We received from the 2 groups 52 (menopause) and 65 (pregnancy) completed questionnaires. The number of woman that use Internet for information about her specific situation was much lower than we thought. The pregnancy-group had a little more interest in consulting Internet for questions about their pregnancy. The difference however was not significant. In other participating European countries (Italy and Spain) the percentage was even lower.

**Conclusion:**

In The Netherlands the number of women that consult Internet for information about pregnancy or menopause is still very limited. One has to keep this in mind when building programs for patient information for the Internet.

